# Cyclic Stretch Induces Cell Reorientation on Substrates by Destabilizing Catch Bonds in Focal Adhesions

**DOI:** 10.1371/journal.pone.0048346

**Published:** 2012-11-12

**Authors:** Bin Chen, Ralf Kemkemer, Martin Deibler, Joachim Spatz, Huajian Gao

**Affiliations:** 1 Department of Engineering Mechanics, Zhejiang University, Hangzhou, People's Republic of China; 2 Engineering Mechanics, Institute of High Performance Computing, A*STAR, Singapore, Singapore; 3 Max-Planck-Institute for Intelligent Systems, Department of New Materials and Biosystems, Stuttgart, Germany; 4 Department of Biophysical Chemistry, University of Heidelberg, Heidelberg, Germany; 5 School of Engineering, Brown University, Providence, Rhode Island, United States of America; University of Ottawa, Canada

## Abstract

A minimal model of cellular mechanosensing system that consists of a single stress fiber adhering on a substrate via two focal adhesions made of catch bonds is adopted to investigate the phenomena of cell reorientation on substrates induced by an applied uniaxial cyclic stretch. The model indicates that the catch bonds in the focal adhesions experience a periodically oscillating internal force with amplitude and frequency controlled by two intrinsic clocks of the stress fiber, one associated with localized activation and the other with homogeneous activation of sarcomere units along the stress fiber. It is shown that this oscillating force due to cyclic stretch tends to destabilize focal adhesions by reducing the lifetime of catch bonds. The resulting slide or relocation of focal adhesions then causes the associated stress fiber to shorten and rotate to configurations nearly perpendicular to the stretching direction. These predicted behaviors from our model are consistent with a wide range of experimental observations.

## Introduction

It has been widely reported that an applied uniaxial cyclic stretch can induce cell reorientation and stress fibers (SFs) realignment on substrates [1]–[15]. In this process, the SFs reorganize and rotate [11]–[13] while cells reorient themselves nearly perpendicular to the stretching direction [1]–[7], [14]. Both the frequency and amplitude of stretch turned out to be important regulating factors of cellular functions and the reorientation process. For example, human osteoblast-like cells exhibit optimal proliferation at the physiological frequency of 1 Hz [8]. For both rat and human fibroblasts at sub-confluent densities, there exists a lower threshold frequency below which no cell reorientation occurs, and the characteristic time of reorientation decreases monotonically as the stretching frequency increases and then saturates at a constant value above 1 Hz [10]. At a fixed frequency, there is also a threshold stretching amplitude for reorientation [2], and the characteristic time decreases almost linearly with the stretch amplitude [10]. The stretch amplitude can also affect the final orientation of cells [2].

The problem of cell reorientation on substrates due to cyclic stretch exemplifies the relatively young but promising field of cellular mechanotransduction, which is currently hindered by a general lack of precise understanding for the essential structural components of cytoskeleton such as stress fibers and focal adhesions (FAs). In spite of this deficiency, there have been rapid progresses in the development of phenomenological but increasingly sophisticated theoretical models for such complex phenomenon [16]–[19]. For example, Na et al. [16] used a constrained mixture model to describe the remodeling of F-actin in response to cyclic stretch. Idealizing a cell as a pair of equal and oppositely directed contractile forces, De et al. [17] assumed that the nearly perpendicular realignment of SFs is caused by a driving force to establish an optimal internal stress and then showed that cell reorientation depends on the stretching frequency. Based on a biochemomechanical model for the formation, dissociation, and contraction of stress fibers, Wei et al. [18] simulated the effect of an applied uniaxial cyclic stretch on the alignment of stress fibers on substrates. Kaunas et al. [19] considered the response of stress fibers to cyclic stretch and found that they behave elastically at high stretch frequencies, but can adjust their reference lengths at low frequencies to maintain tensional homeostasis [20], [21]; the process of cell reorientation was then regarded as a consequence of increasing rates of disassembly of stress fibers under high strain rates, resulting in an accumulation of stress fibers in orientations that avoid rapid changes in length. Although these models have revealed significant insights in the process of cell reorientation under cyclic stretch, a number of important observations remain unexplained [10]: Why do cells become rounded at the beginning of reorientation? Why is the physiological frequency of 1 Hz so special for various cell types? Why does the characteristic time of reorientation decrease with the cyclic amplitude at a fixed stretching frequency? Why is there a lower threshold frequency below which no reorientation occurs? Finally, why is there a saturation frequency around 1 Hz beyond which the characteristic time of reorientation no longer changes with the stretch frequency. To address these questions, we note that a common deficiency of all existing models is that the role of FAs has been completely neglected, even though FAs sliding, RhoA activation and actomyosin contraction have been identified as the key players in cellular reorientation under cyclic stretch [22]. There is currently no model capable of including all these key components in an integrated framework. The main objective of the present work is to start filling this gap by providing the first integrated model of cell reorientation, which is concise but still capable of capturing the essential physics of the problem, so that the big picture of the biophysical process can be subject to further investigations.

**Figure 1:**
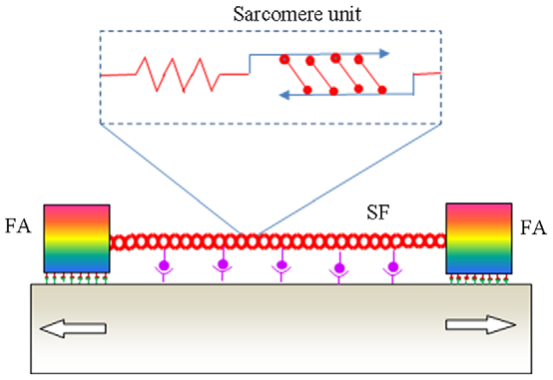
The elastosarcomere-adhesion model of a stress fiber adhering on a substrate via two focal adhesions of catch bonds and distributed anchoring linkages along the stress fiber. The activated sarcomere units in the stress fiber shorten or lengthen following the linearized Hill's law, while the focal adhesions can slide or relocate in response to an applied cyclic stretch.

Our model is based on recent finding that α_5_β_1_ integrin clusters play a dominant role in providing the mechanical strength of FAs [23]. Interestingly, it has been shown that the bonding between an α_5_β_1_ integrin and its ligand is a catch bond [24], similar to those between bacterial adhesive proteins and mannose [25], between P-seletin and its ligand [26], between L-selectin and its ligand [27], and between myosin and actin [28]. Catch bonds are considered critical for cells to stabilize adhesion [29]. To reveal the essential physics of cell reorientation under cyclic stretch, here we adopt a minimal model of cellular mechanosensing system, hereafter referred to as an elastosarcomere-adhesion (ELSA) model, which integrates the dynamic behaviors of a stress fiber adhering on a substrate via two focal adhesions made of catch bonds, as schematically shown in Fig. 1. In this model, the passive elasticity of a sarcomere unit in the stress fiber is accounted for by a spring constant *k* while the active actomyosin contractility is assumed to obey a linearized Hill's law. Although the exact relation between the force and stretching velocity of stress fibers is not yet available, here we adopt the linearized Hill's law for the active response of a sarcomere unit for the following reasons: (1) There is significant structural similarity between a stress fiber and a skeletal muscle fibril for which Hill's law was originally deduced [30]; (2) The linearized Hill's law can be regarded as a first order description of the essential properties of a sarcomere. We will show that the behaviors of the ELSA model under cyclic stretch are consistent with a wide range of experimental observations for cell reorientation on substrates.

## Analysis

### Localized versus homogeneous activation modes of stress fiber

The non-muscle myosin II motors are responsible for the active contractility of a stress fiber which typically has length of 50 µm [18], [31]. With an average unit length of sarcomeres around 1 µm [18], there should be about 50 sarcomere units in a SF. However, the shortening or lengthening of a SF can be quite non-uniform along its length [32]. Under cyclic stretching, there exist evidences for a transition between a localized activation mode of SF at low stretching frequencies to a homogeneous activation mode at high stretch frequencies. In the localized activation mode, only one or a few sarcomere units in the stress fiber are activated at any given time, while in the homogeneous activation mode, all or a large majority of sarcomere units are simultaneously activated. Although the detailed mechanisms of localized versus homogeneous activation modes need to be further clarified, we can identify a number of experimental observations that strongly suggest this behavior. First, it was found that the myosin concentration is usually higher in the peripheral regions while the α-actinin concentration is higher in the central regions of the cytoskeleton, and the peripheral regions of a SF usually shorten while the central regions stretch [32]. Second, under high frequency cyclic stretch, it has been observed that significant remodeling occurs in the central regions of the cytoskeleton [33] and at the same time RhoA activity increases dramatically, indicating more activated myosin activities throughout the cytoskeleton [22]. These provide indirect experimental evidences for homogeneous activation of sarcomere units along a stress fiber in the high frequency regime.

Furthermore, we can provide a theoretical argument why there should be a transition between localized and homogeneous activation modes of a stress fiber as the cycling frequency increases. Recent laser nanosurgery experiments [34] have suggested direct mechanosensing between ventral stress fibers and substrate due to multiple localized anchor points along the actin bundles. These localized anchor points, as schematically shown in Fig. 1, are expected to have intrinsic relaxation time scales much shorter than mature focal adhesions. At low stretch frequencies, the interaction forces between the stress fiber and substrate via the localized anchor points could be fully relaxed due to bond rupturing and rebinding. However, at sufficiently high frequencies, there may not be sufficient time for these bonds to relax during the stretching half-cycles. As a result, the whole stress fiber would be stretched directly by the substrate via these anchoring points, which would lead to homogeneous activation of all or a large majority of the sarcomere units. It is very possible that this is the main cause for the experimentally observed remodeling of SFs and dramatic increase in RhoA activity at high stretching frequencies [22].

### Two intrinsic clocks of stress fiber

**Figure 2:**
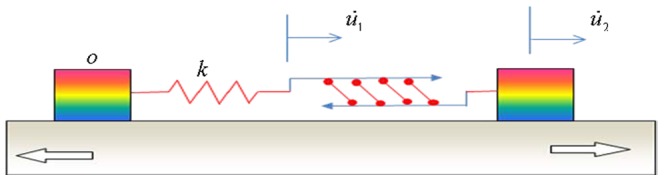
A sarcomere unit of stress fiber anchored on a substrate under high frequency cyclic stretch. At low cyclic frequencies, the distributed anchoring points are released so that the sarcomere unit should be replaced by the entire stress fiber anchored on two focal adhesions.

With the basic model depicted in Fig. 1, we can show that under cyclic stretch the force in the SF and focal adhesions is controlled by two intrinsic clocks of the stress fiber. First, let us consider the homogeneous activation mode where all sarcomere units are simultaneously activated. In this case, we consider a typical sarcomere unit with spring constant *k* and isotonic load 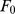, which is directly subject to cyclic stretch from the substrate, as schematically shown in Fig. 2. The spring constant represents the passive elastic property of the sarcomere, with the following force-stretch relation
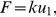
(1)where 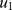 is the elastic displacement of the sarcomere as it shortens or lengthens.

The active contractility of the sarcomere is modeled by the linearized Hill's law with the following force-velocity relation [18], [30],
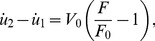
(2)where 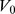 denotes a reference velocity and 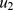 is the relative displacement of the substrate between two adjacent anchoring points. An applied cyclic stretch on the substrate is then represented as
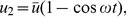
(3)where 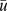 is the stretching amplitude and 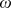 the angular frequency of stretch. Combining Eqs. (1–3) yields the following equation which governs the force variation in the sarcomere,
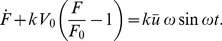
(4)
Eq. (4) can be solved in closed form as
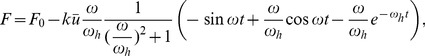
(5)where
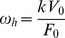
(6)is identified as the characteristic frequency associated with the homogeneous activation mode of a stress fiber. This shall be referred as the *upper intrinsic clock* of the stress fiber. Equation (5) is obtained by explicitly solving the first-order differential equation Eq. (4) subject to the initial condition that 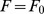 at 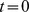. Note that no assumption is necessary in deriving Eq. (5) from Eq. (4).

**Figure 3:**
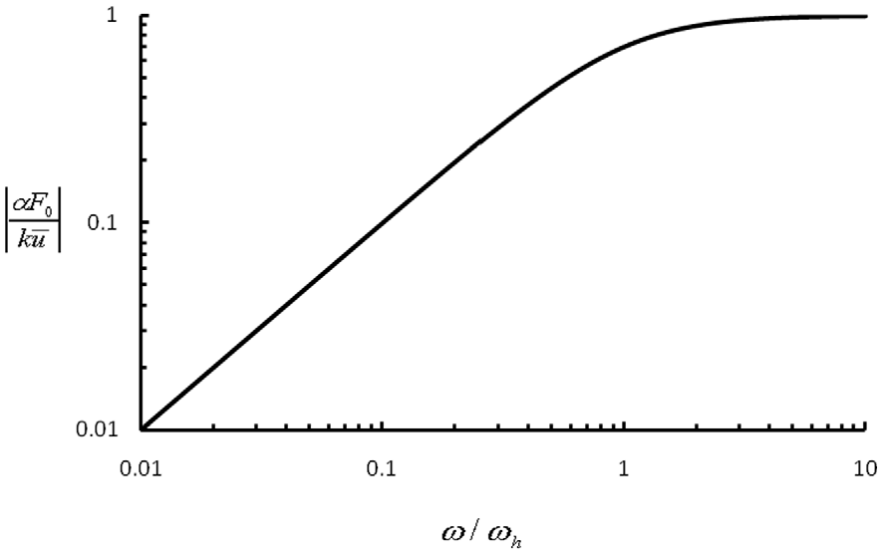
Amplitude of steady state force oscillation in a homogeneously activated stress fiber as a function of the cyclic stretching frequency. Note the saturation of force variation beyond the intrinsic clock frequency 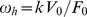. Similar behavior would occur in a locally activated stress fiber with the lower intrinsic clock frequency 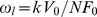 where 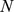 is the total number of sarcomere units in the stress fiber.

Equation (5) indicates that, at steady state, the force in the sarcomere oscillates periodically about the isotonic load with frequency 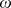 and normalized amplitude (with respect to 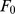),
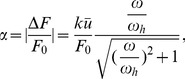
(7)which depends on both the stretching amplitude and frequency, as shown in Fig. 3. Note that the square root in Eq. (7) is related to the norm of the first two terms within the parenthesis in Eq. (5).

Similar analysis of force variation could be carried out for the localized activation mode of a stress fiber. In this case, only a single sarcomere unit is activated at any given time. The ELSA model shown in Fig. 2 remains valid except that the spring constant *k* of a single sarcomere unit should now be replaced by that of the entire stress fiber, which is equal to
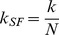
(8)where *N* is the total number of sarcomere units in the SF. This then gives rise to a characteristic frequency associated with the localized activation mode of a stress fiber,
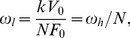
(9)which shall be referred to as *the lower intrinsic clock* of the stress fiber.

A SF typically has a tension modulus of 50 nN (corresponding to a spring constant of 1 pN/nm) [18], [31], length of 50 µm and 50 sarcomere units with isotonic load of 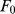
∼2 nN. Note that the elasticity of FAs has been neglected in the above force analysis, in view of the low spring constant of SF. These parameters lead to 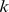
∼50 pN/nm. At low stretching frequencies, we assume that the SF is in the localized activation mode, i.e. with actomyosin activities focused in the sarcomere units around the peripheral regions of the cytoskeleton, with 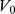
∼30–300 nm/s [18], [35]. This then transitions into the homogeneous activation mode at very high stretching frequencies. Taking 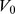
∼30–300 nm/s, 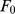
∼2 nN, 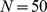 and 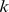
∼50 pN/nm, we estimate that the two intrinsic clocks of stress fiber have the following characteristic cyclic frequencies
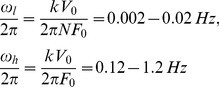
(10)The observed threshold frequency for cell reorientation is 0.01 Hz for rat fibroblasts and 0.1 Hz for the human fibroblasts [10], which are close to 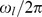. This suggests that the localized activation mode of stress fiber may indeed govern the reorientation behavior in the low frequency regime, with a lower threshold frequency to regulate cell reorientation on substrates. In other words, there exists a minimum stretching frequency for cell reorientation as long as there is at least one activated sarcomere unit attempting to maintain force homeostasis in the SF and focal adhesions (see discussions in the next two sections). On the other hand, 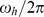 is close to the experimentally reported saturation frequency around 1 Hz beyond which the characteristic time of cell reorientation no longer changes [10]. This is consistent with our assumption that the homogeneous activation mode of stress fiber governs the reorientation behavior in the high frequency regime. In this case, the rate of cell reorientation should be limited by the time scale for sarcomere units to shorten or lengthen simultaneously, as this is the fastest response of a sarcomere to a rapidly varying external load.

Since 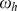 is the intrinsic property of a sarcomere unit of stress fiber, it can be conserved for various cells as long as the average properties of sarcomere units are preserved. Note that Eq. (7) indicates that the amplitude of force variation is close to the saturation value around 1 Hz when 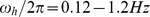 (Fig. 3). Without specification, we set 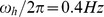 which falls in this range in the following analysis.

### Size of focal adhesions under cyclic stretch

Focal adhesions may grow or shrink under an external load. Since cells are known to maintain a constant stress on FAs on a relatively stiff matrix [36], the size of an FA should be proportional to the applied force. An FA mainly grows along the axial direction of its associated SF and a mature FA is rather slender. Therefore, the FA size can also be described in terms of its length. Under dynamic loading, the response of FAs should involve a characteristic time for signal transduction. As a first order approximation, the size of FAs at time *t*, denoted as 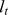, can be expressed as
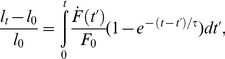
(11)where 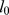 is a reference length and 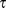 is a characteristic time on the order of 3–90 minutes [10], [37] of FAs in response to a changing applied load. Assume that a constant force of magnitude 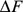 is applied at time 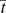, we have 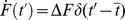, where 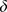 denotes the Dirac delta function. In this case, Eq. (11) shows that 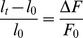 as 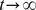, indicating that the size of a FA is proportional to the applied force so that the stress within a FA would remain a constant value, in consistency with the experimental finding [36].

According to Eqs. (5–7, 11), it can be shown that upon cyclic stretch
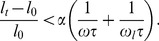
(12)Note that 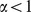 when the stretch amplitude is less than 10%. Eq. (12) indicates that there should be very limited focal adhesion growth (<18%) along the direction of stretch in the frequency regime 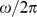
>0.01 Hz when 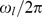 = 0.01 Hz.

### Lifetime of catch bonds under cyclic stretch

The dissociation lifetime of a catch bond increases with force when the force is relatively small. However, under very large forces, the lifetime of bonds should always decrease, irrespective of the bond type. Under an increasing applied force, the lifetime of a catch bond typically increases first, reaches a peak value and then decreases (24, 26). A question of interest here is how the cyclic stretch would affect the lifetime of such a catch bond. Since there is only limited change in the size of FAs and the force within the SF essentially oscillates periodically about its isotonic value, the force on each catch bond, 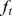, should oscillate periodically about an optimal value 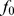 corresponding to the longest bond lifetime, with the following form
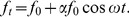
(13)The bond breaking rate 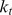 of the catch bond is expected to reach a minimum at 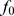 and rise whenever the applied force deviates from 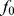. In a quadratic approximation near 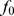, this behavior can be expressed as
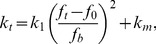
(14)where 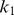 is a constant and 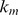 is the minimum bond breaking rate at 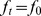. The average bond lifetime is then calculated as [38]

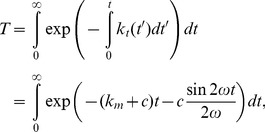
(15)where 
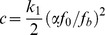
.

It has been shown that an integrin catch bond can switch from a state of high detachment rate to one of low detachment rate upon activation [24]. Here we neglect the state of high detachment rate which should play rather minor roles in a mature FA. Consequently, it is noted that the bond lifetime at the low detachment rate might be longer than the experimentally reported value. Also, the reported lifetime beyond 30 pN should be a lower limit because it was caused by Fc–GG-7 dissociation instead of FNIII7–10–α5β1-Fc dissociation [24]. Accordingly, we take 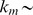
0.1 Hz [24]. Since the bond force induced by cyclic stretch varies slightly around 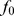, 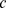 could be comparable to or smaller than 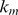. For 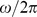
>0.3 Hz, 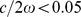 and we find
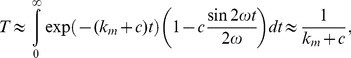
(16)which indicates that cyclic stretch will reduce the lifetime of a catch bond. Note that a constant bond force,
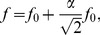
(17)would result in the same bond lifetime as that given in Eq. (16) and, therefore, could be regarded as an effective force induced by the cyclic stretch. Note that, for simplicity, we have assumed that the catch bonds are aligned with the axial directions of the SF in deriving Eqs. (13–17).

### Rotation velocity of stress fiber

When the force in each catch bond is close to the optimal value 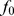, the focal adhesion is expected to be most stable. Under cyclic stretch, the bond force oscillates periodically about 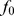 so that the bond lifetime is reduced according to Eq. (16). We postulate that the less stable FAs would slide or relocate to more stable configurations (see Movie S1 in the supplemental Information).

**Figure 4:**
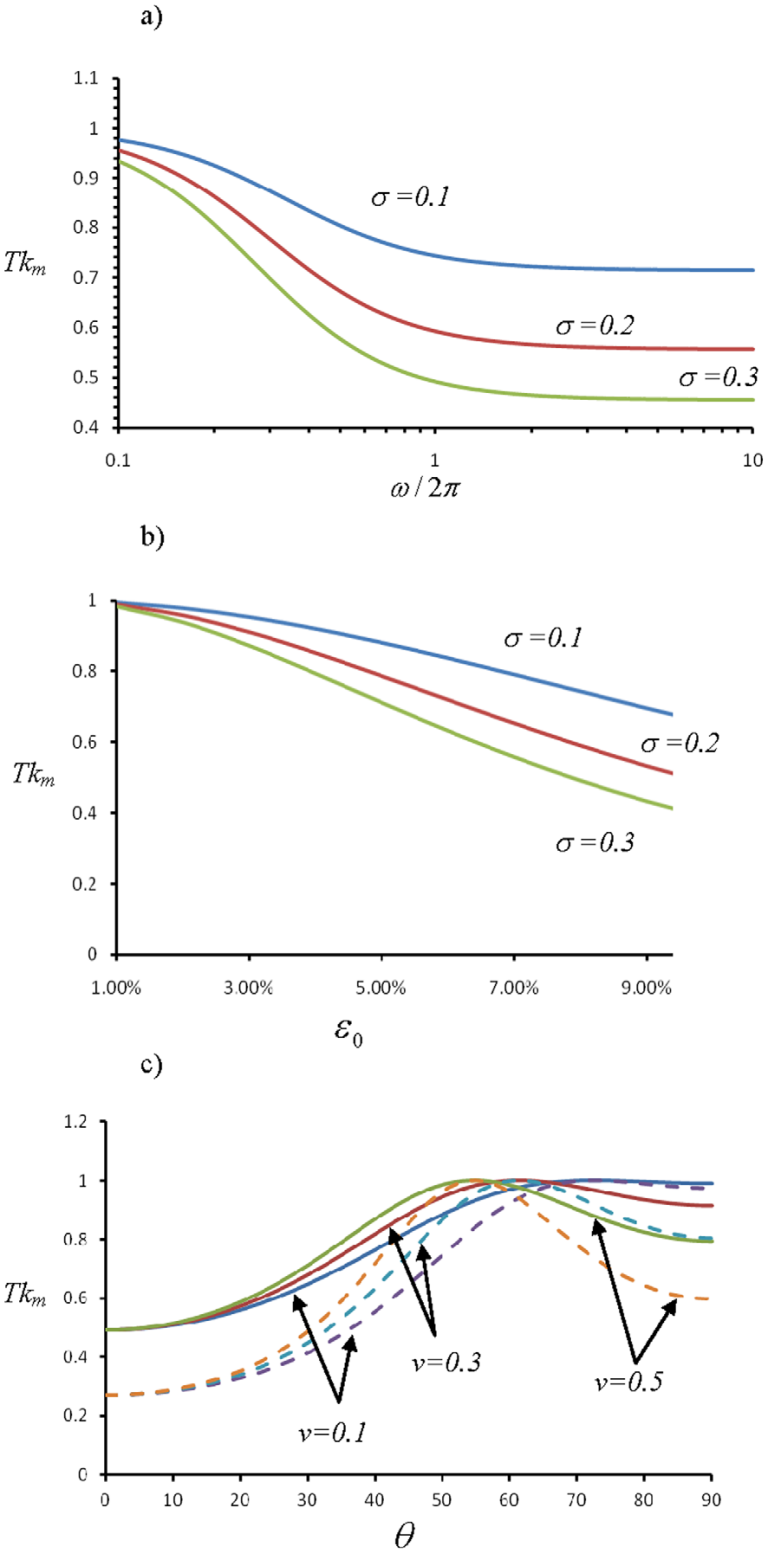
Dependence of average lifetime of catch bond on a) cyclic frequency, b) cyclic amplitude, c) stress fiber orientation. The parameters 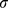 and 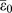 are taken to be 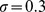, 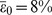 for solid lines and 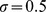, 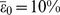 for dashed lines. Other parameters are 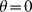, 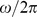 = 1 Hz and *v* = 0.5. Homogeneous activation of stress fiber is assumed in these calculations.

It can be shown that the stretch displacement amplitude in an SF of length 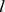 and orientation angle 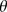 is
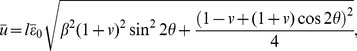
(18)where 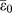 is the amplitude of cyclic strain in the elastic substrate at 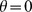, 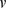 is Poisson's ratio, and 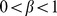 reflects possible contribution from the resolved shear strain (see Appendix S1 for derivations). Here we assume 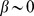 and focus on the homogeneous activation mode of stress fiber. In this case, we note that 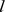 should be replaced by the length of a sarcomere unit. It follows from Eqs. (7, 16, 18) that the average lifetime of catch bonds is
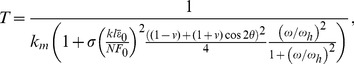
(19)where 
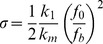
. The dependence of *T* on 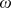, 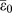, and 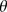 is plotted out in Fig. 4. In Fig. 4a, the bond lifetime *T* is shown to decrease with 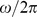 until ∼1 Hz, beyond which it saturates to a constant. In Fig. 4b, *T* is shown to decrease monotonically with 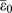. These results are qualitatively consistent with the reported behaviors of the characteristic time of cell reorientation upon cyclic stretch [10]. Figure 4c shows that in order to prolong *T*, the stress fiber would favor an orientation nearly perpendicular to the stretch direction. The exact angle of the equilibrium configuration, however, depends on Poisson's ratio *v* [4]. Note that Eq. (19) is for the average lifetime of a single catch bond, which can be quite different for a cluster of catch bonds within a focal adhesion. As such, it cannot be used to model the power-law decrease of the characteristic time of re-orientation as frequency is increased, as observed in experiments (10). While it will be interesting to simulate the dependence of the average lifetime of a cluster of catch bonds on the cyclic frequency, we prefer to leaving such a refined study in future investigations because the molecular details of a focal adhesion are not completely clear at this stage.

In deriving Eq. (19), we have assumed homogeneous activation of all sarcomere units along the stress fiber. In principle, this is valid only at high frequencies. While this simplification is not expected to affect the predictions from Eq. (19) at high frequencies, ideally the behaviors at low to intermediate frequencies should be more rigorously addressed by taking into account the stochastic behaviors of distributed anchoring bonds along the stress fiber. This, however, is currently hindered by the lack of sufficient quantitative information about the detailed interactions between SF and substrate. Further experiments will be needed before progresses can continue in this direction.

Since the product 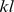 is the (length-independent) intrinsic tension modulus of stress fiber, Eq. (19) indicates that the bond lifetime is independent of the length of SF. Therefore, while the sliding of FAs leads to shortening of SFs and rounding of cells, as observed in experiments [10], the contraction velocity of SFs is not expected to change. The shortened, less flexible SFs should be increasingly constrained by their neighbors as well as by the cell nucleus, affecting its orientation and aspect ratio [39]. These constraints may have prevented the SFs from shrinking indefinitely. On the other hand, Eq. (19) indicates that the focal adhesions would become more stable if the SFs could rotate, as seen from Movie S1 in the Supplemental Material. The reason is that the stretching displacement amplitude is reduced with increasing 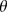 and reaches the minimum at the following most preferred angle
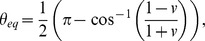
which is equal to 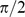 only when 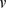 = 0 [40].

To understand the dependence of the rotational velocity of SFs on 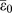 and *f*, here we employ a simple transition-state model. Suppose that state “2” of FAs is more stable than state “1” and FAs could transit from state “1” to state “2” by a very small orientation change, 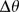, at the following net rate:
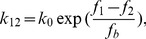
(20)where 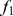 and 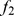 are effective bond forces at states “1” and “2”, respectively, and 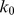 is a reference rate. The transition velocity can be expressed as
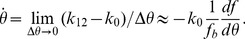
(21)Using the expression for the effective force due to cyclic stretch given in Eq. (17), we obtain
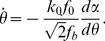
(22)Neglecting the Poisson effect and assume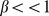, this leads to the rotation velocity of SFs as
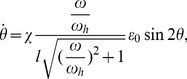
(23)where 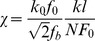. Equation (23) indicates that shorter stress fibers should rotate faster.

## Discussion

Reorientation of cells on substrates subjected to cyclic stretch has been widely observed and often regarded as a natural reaction of focal adhesions and stress fibers in response to cyclic loads [14]. Since the clustering of α_5_β_1_ integrins, which form catch bonds with their ligands [24], determines the strength of FAs [23], it is important to investigate the role of catch bonds in this phenomenon. Without the applied stretch, the cellular mechanosensing system are considered to be at a homeostatic state, with forces in the SFs maintained near the isotonic load and those in the catch bonds within FAs at the optimal load corresponding to the longest lifetime.

Under an applied cyclic stretch, our elastosarcomere-adhesion model shows that the force within the SF oscillates about the isotonic load. The amplitude of this force oscillation increases with the amplitude of the cyclic strain, and also with the stretch frequency until it saturates at a critical frequency [18]. In this model, the non-muscle myosin IIs within a stress fiber are assumed to actively resist stretching according to the linearized Hill's law. We emphasize that the exact dependence between the force and the sliding velocity for a stress fiber is not yet fully understood at the present time due to the inherently dynamic nature of SFs. We have adopted the linearized Hill's law for the active response of a stress fiber for the following reasons: 1. There is significant structural similarity between a stress fiber and a skeletal muscle fibril for which Hill's law was originally deduced [30]; 2. The linearized Hill's law can be regarded as a first order description of the essential behaviors of a sarcomere.

In our model, it has been assumed that the stress fiber follows a localized activation mode in the low cyclic frequency regime and a homogeneous activation mode in the high cyclic frequency regime. The localized activation mode is motivated by the observations that myosin concentration is higher in the peripheral regions while α-actinin concentration is higher in the central regions, and that the peripheral regions of a SF usually shorten while the central regions usually stretch [32]. The homogeneous activation mode is motivated by the observation that there is significant remodeling and more activated myosins in the central regions at high frequencies [33]. It follows from these assumptions that the amplitude of force variation within the SF is regulated by two intrinsic clocks, one associated with the localized activation mode and the other associated with the homogeneous activation mode of SF. The lower intrinsic clock sets a lower threshold frequency for cell reorientation and the upper intrinsic clock sets a saturation frequency (around 1 Hz) beyond which the characteristic time of reorientation no longer changes with the cycling frequency. Since the upper intrinsic clock is independent of the length of stress fiber, it can be conserved for various cells as long as the average properties of sarcomere units are preserved. The hypothesis that a ventral SF would become more uniformly adhered to the substrate at high stretching frequencies, while a natural consequence of the experimentally suggested multiple localized anchor points along the actin bundles [34], should nevertheless be regarded as an assumption that requires direct experimental confirmation.

It is further shown that the focal adhesions essentially maintain their sizes under an applied cyclic stretch due to the much larger characteristic time scale associated with FA growth/shrinkage, which is consistent with the observation that massive FA rearrangements under cyclic stretch were accomplished by sliding instead of de novo formation of FAs in the initial process [22]. Under this condition, the force in the catch bonds would oscillate periodically about the optimal load and, as a consequence, the bond lifetime decreases with increasing stretching amplitude as well as with increasing stretching frequency until a critical value. Note that the effect of a periodic force on the lifetime of catch bonds has been investigated in the literature under different circumstances [41], [42]. Since an applied force can in principle strengthen and mature FAs even in the absence of catch bonds, there could be alternative physical interpretations for the catch-bond-like behaviors of FAs investigated here.

Our analysis suggests that there exists a lower threshold frequency for cell reorientation due to a lack of force variation within the stress fibers at very low frequencies, in consistency with the corresponding experimental observations of no stress fibers disassembly or rotation and no significant cell reorientation at sufficiently low frequencies [10]. This suggests the importance of a lower intrinsic clock associated with the localized activation mode of stress fiber. Here we point out that some experiments showed fibroblasts aligned parallel to the stretch direction in a collagen matrix upon quasi-static loading [43]. This difference might be due to the contact guidance of mechanically aligned collagen fibers on cells [10]. Also, in endothelial cells (ECs) treated with Rho-kinase inhibitor Y27632, actin fibers were oriented parallel to the stretch direction at high frequencies [44]. We suspect that this difference may have arisen because SFs formation and contractility were impaired by Y27632, in consistency with an analysis suggesting that thick actin fibers were formed through the bundling of thinner fibers oriented toward the stretch direction [44]. Our analysis also suggests that reorientation does not occur under isotropic, equibiaxial cyclic stretch [16]. In addition, according to Eq. (18), the final angle of reorientation corresponding to the smallest amplitude of the cyclic stretch is not always 90° and generally depends on the Poisson's ratio of the substrate, in agreement with the experiments [39].

We have employed a transition-state model to estimate the rotation velocity of SFs upon cyclic stretch. Although this transition-state model is very simple, it is also quite general and does not assume any specific mechanism for FAs translocation. For example, in the case that bonds on one side of an FA may be more stable than those on the other side, causing the FA to slide slowly, the transition-state model needs only to be specifically interpreted as describing a characteristic time scale associated with this slow sliding process. This model is presented in this simple way because the molecular details in an FA is not yet fully clear and it is therefore better to leave a refined study to further investigation in the future. In a recent paper, we have observed a dislocation-like friction-slippage pattern for slip bonds [45], and it seems quite possible that similar movement can be applicable for catch bonds as well. Our analysis shows that the lifetime of catch bonds decreases with the amplitude of the cyclic strain and frequency, reaching saturation when the latter exceeds 1 Hz. Accordingly, the characteristic time of reorientation is expected to decrease with increasing loading frequency before saturating around 1 Hz (which is shown to be an intrinsic property of sarcomere under cyclic stretch) and to decrease with the amplitude of the cyclic strain within the elastic substrate. These results are all in consistency with the experimental observations [10].

Previously, considering cells as force dipoles, De et al. [17] developed a model with frequency dependence of the characteristic time of reorientation, including both power law at low cyclic frequencies and the saturation at high frequencies [10]. However, the model of De et al. predicts that cells should align in parallel with the stretch direction in the limit of very low frequencies, which appears to be inconsistent with experimental observations [10]. In addition, the model by De et al. is a phenomenological model that does not involve any structural information of SFs or FAs. Kaunas et al. [19] focused on the roles of myosin II in cellular reorientation under cyclic stretch with a sarcomeric model of SFs and showed that the SFs can maintain a constant level of tension at low cyclic frequencies through myosin sliding but they tend to behave elastically at very high cyclic frequencies [19]. While Kaunas et al. focused on the dependence of SF reorientation on cyclic frequency, they did not consider the roles of FAs. Our present work built upon these previous studies and extend them with an integrated model that accounts for the behaviors of both FAs and SFs during cellular reorientation under cyclic stretch. Our analysis indicates that the catch bonds in FAs experience an oscillating force with amplitude and frequency controlled by two intrinsic clocks. This oscillating force tends to destabilize catch bonds in the FAs and causes the associated SFs to shorten and rotate.

**Figure 5:**
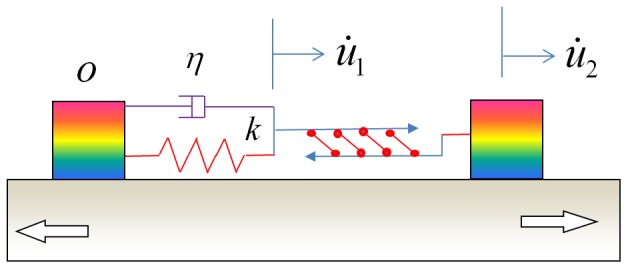
A sarcomere unit of SF with passive viscosity anchored on a substrate under high frequency cyclic stretch. At low cyclic frequencies, the distributed anchoring points are released so that the sarcomere unit should be replaced by the entire SF anchored on two FAs.

It has been previously reported that SFs can be inherently viscoelastic [46]. This effect can be modeled by introducing an additional dashpot with viscous coefficient 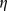 in the sarcomere unit, as schematically illustrated in Fig. 5. With this modification, the force within the SF is described by
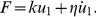
(24)Combing Eqs. (2–3, 24), we obtain the modified force profile within the SF as
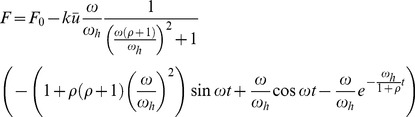
(25)where 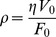 and we have imposed the initial condition that 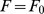 at 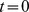. Equation (25) indicates that, in the viscous sarcomere model, the force in the SF is still oscillatory, but the normalized amplitude of force variation (with respect to 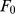) depends on the parameter 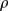. When 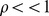, Eq. (25) degenerates to Eq. (5). If we assume that the viscosity of stress fiber is similar to that of blood at 37° and the size of stress fibe is ∼50 µm, we would have 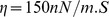 and a very small 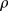
∼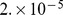. In this case, the effect of viscosity would be negligible. On the other hand, if we assume 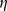 to be comparable to that of honey, in which case 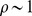, the inherent viscosity of SFs should have a strong effect. For example, if 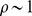, Eq. (25) predicts that the amplitude of force variation approaches infinity when 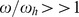. Currently, the exact value of 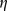 for a SF is not available. Since the inhibition of active tension generation was found to significantly reduce the initial rate of retraction [46] and the time scale of SF retraction observed by Kumar et al. [46] is about 5 s, which is surprisingly similar to that reported by Russell et al. [35], one wonders if the observation of Kumar et al. [46] was in fact induced by active retraction rather than passive viscous dissipation.

**Figure 6:**
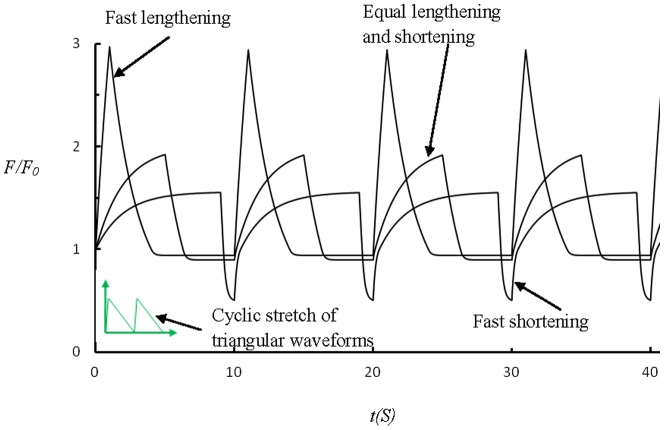
Force profiles within a SF under cyclic stretches of triangular waveforms at stretching frequency of 0.1 Hz and amplitude of 10%. The lengthening and shortening velocity constants are taken to be 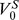 = 200 nm/s, and 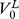 = 20 nm/s, respectively. The results clearly demonstrate that faster lengthening leads to much stronger oscillation in the force within the SF.

Recently, Todon et al. [47] showed experimentally that stretching affects reorientation of stress fibers more significantly than relaxation. To understand this effect, we assume that the shortening speed of a SF is higher than its lengthening speed in the vicinity of the isotonic force, similar to the behavior of skeletal muscle [48]. In this case, Eq. (2) should be replaced by
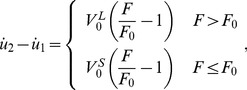
(26)where 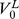 and 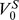 are two velocity constants with 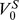 generally larger than 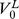. Together with Eq. (1), we can study the effect of cyclic stretch with triangular waveforms, as in Todon et al. [47]. The resulting force profiles in the SF are plotted out in Fig. 6, which shows that a high applied lengthening rate leads to much larger forces within SFs than a high applied shortening rate. This implies that, at the same strain rate, lengthening should be more effective in destablizing catch bonds, which can explain the observed asymmetric effect that SFs are more responsive to the lengthening rate than the shortening rate [47]. Note that this phenomenon was attributed to force-induced asymmetric bond breaking rates although it was not clear which specific bonds were affected [47].

Although we have only considered catch bonds within FAs, catch-bond-like behaviors could also exist elsewhere in the pathway for stress generation and transmission, which may have contributed to cellular reorientation in response to periodic stretches even at very low frequencies [49]. It was reported that, in response to a transient stretch of 4 second duration, the cytoskeleton of various cells promptly fluidized over a time scale of a few seconds, followed by re-solidification in a few minutes [50]. It was further demonstrated that the fluidization trumped the localized reinforcement response [51] in loading conditions expected in most physiological circumstances. Through comprehensive measurement, Chen et al. [52] proposed that the extent of the fluidization response was localized to the relaxation phase in a manner suggesting cytoskeletal catch bonds. When applying periodic stretches at a very low frequency ∼0.02 Hz, Krishnan et al. [49] found that cytoskeleton promptly fluidized, followed by slow re-solidification response along the axis perpendicular to the imposed stretch.

Note that the results by Krishnan et al. [49] or Chen et al. [52] are apparently at odds with those by Todon et al. [47], where SFs were found to be much more responsive to the stretching rate than the relaxation rate upon cyclic loading of asymmetric waveforms. Specifically, Todon et al. [47] found that fast stretching rate at 2 Hz induced cell reorientation while fast-relaxation rate at 2 Hz did not. Also, the fluidization and re-solidification mechanism suggested by Krishnan et al. [49] is not supported by Movie S1 in the Supplementary Material of the present paper either, which directly demonstrates rotation of SFs away from the direction of the applied stretch, as well as some fusion of short fibers under a cyclic stretch amplitude of 8% [53].

We suspect these apparent discrepancies may have been caused by the effect of substrate stiffness. The substrate stiffness in the papers reporting cytoskeleton fluidization is generally on the order of KPa [49]–[52], which is much lower than that of MPa used either by us or Todon et al. [47]. It is known that substrate stiffness can strongly influence the phenotype of adherent cells [54], [55] and stiffer substrates lead to stronger FAs [56]. Thus, it might be possible that the mechanisms proposed in the present paper for cellular reorientation upon cyclic stretch dominates on relatively stiff substrate with relatively low stretch amplitude (<10%) while fluidization-induced cell reorientation dominates on soft substrate with relatively high stretch amplitude (>10%). Indeed, Krishnan et al. [49] reported that there was very limited cytoskeleton fluidization upon cyclic stretch with amplitude of 5%. It should be emphasized that the underlying mechanism for cytoskeleton fluidization is not yet clear and one might wonder if the physical connections between FAs and SFs might be partially damaged due to the transient stretch that leads to the liquidization of cellular cytoskeleton, since the time scale of re-solidification is close to that of the formation of FAs.

The present model is overly simplistic in a number of ways. The key assumptions of the model include localized activation mode of SFs at low cyclic frequencies and homogeneous activation modes of SFs at high cyclic frequencies, that the ends of SFs have the same velocity as the adhered substrate, that the strength of a FA is mainly determined by catch bonds, that the directions of catch bonds can be modeled as parallel to the axial directions of SFs, and that the less stable FAs would cause the associated SFs to shorten or rotate to more stable configurations. These assumptions need to be validated by further experiments. In addition, the complex biochemical signaling process such as the interplay between Rho pathway activity and stretching amplitude [57] and frequency have not been considered. We have not considered the remodeling of stress fibers during the reorientation process. Focal adhesions are more than just a cluster of catch bonds and how they exactly slide is not yet clear. The behavior of stress fiber may not strictly follow the linearized Hill's law. The interactions among multiple stress fibers, focal adhesions, microtubules, nucleus, and other subcellular and cytoskeletal components have not been considered in this minimal model. The formation of new focal adhesions may also play a role in the process. Also, only ventral SFs are considered in the present model. In spite of all these shortcomings, it is encouraging that such a minimal model can predict behaviors that are in broad agreements with virtually all existing experimental observations.

## Conclusion and Outlook

We have investigated the role of catch bonds in cellular reorientation under cyclic stretch. Our analysis shows that the force within the catch bonds oscillates periodically around its optimal value, with amplitude and frequency regulated by two intrinsic clocks of the stress fiber, one associated with localized activation and the other with homogeneous activation of sarcomere units along the stress fiber. The amplitude of the force variation increases with both the stretch amplitude and the frequency, and approaches a saturation value when the latter exceeds the upper intrinsic clock frequency of stress fiber around 1 Hz. Our analyses indicate that the larger the amplitude of force variation within the catch bonds, the less stable the FAs become. The destabilized FAs would then slide or relocate and lead the associated SFs to contract or rotate toward the most stable configurations, in which the amplitude of force variation could be minimized. Together, this study suggests the critical roles of catch bonds in cell reorientation induced by cyclic stretch.

Our model predictions suggest further experimental investigations in this area. For example, an image-correlation-based tracking method can be used to measure the change in length of the sarcomeres during stress fiber retraction [35]. Stress fiber FRAP experiments [34] combined with cyclic stretch could support the idea that SFs adopt different activation modes depending on the stretch frequency. It is possible to study FA dynamics in details by transfecting cells with a GFP-vinculin construct and tracking them in time-lapse fluorescence microscopy [22]. It is also possible to remove catch bonds in focal adhesion with micelle nanolithography to fabricate nanoscopically controlled biomolecule anchors that are subsequently transferred on elastic polymers. Such experiments would further help reveal the underlying mechanisms of FAs and SFs behaviors under cyclic stretch.

## Supporting Information

Figure S1
**Schematic of a stress fiber at an angle** 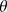 **with respect to the direction of cyclic stretch.**
(TIF)

Appendix S1 **Dependence of cyclic stretch amplitude of the SF on the orientation angle.**
(DOCX)

Movie S1 **SFs shortening/rotation upon cyclic stretch.**
(AVI)
