# Supplementary figures and images for: Cyclic Stretch Induces Cell Reorientation on Substrates by Destabilizing Catch Bonds in Focal Adhesions
Source: PLoS One. 2012 Nov 12;7(11):e48346. doi: 10.1371/journal.pone.0048346 (PMC3495948; doi:10.1371/journal.pone.0048346)

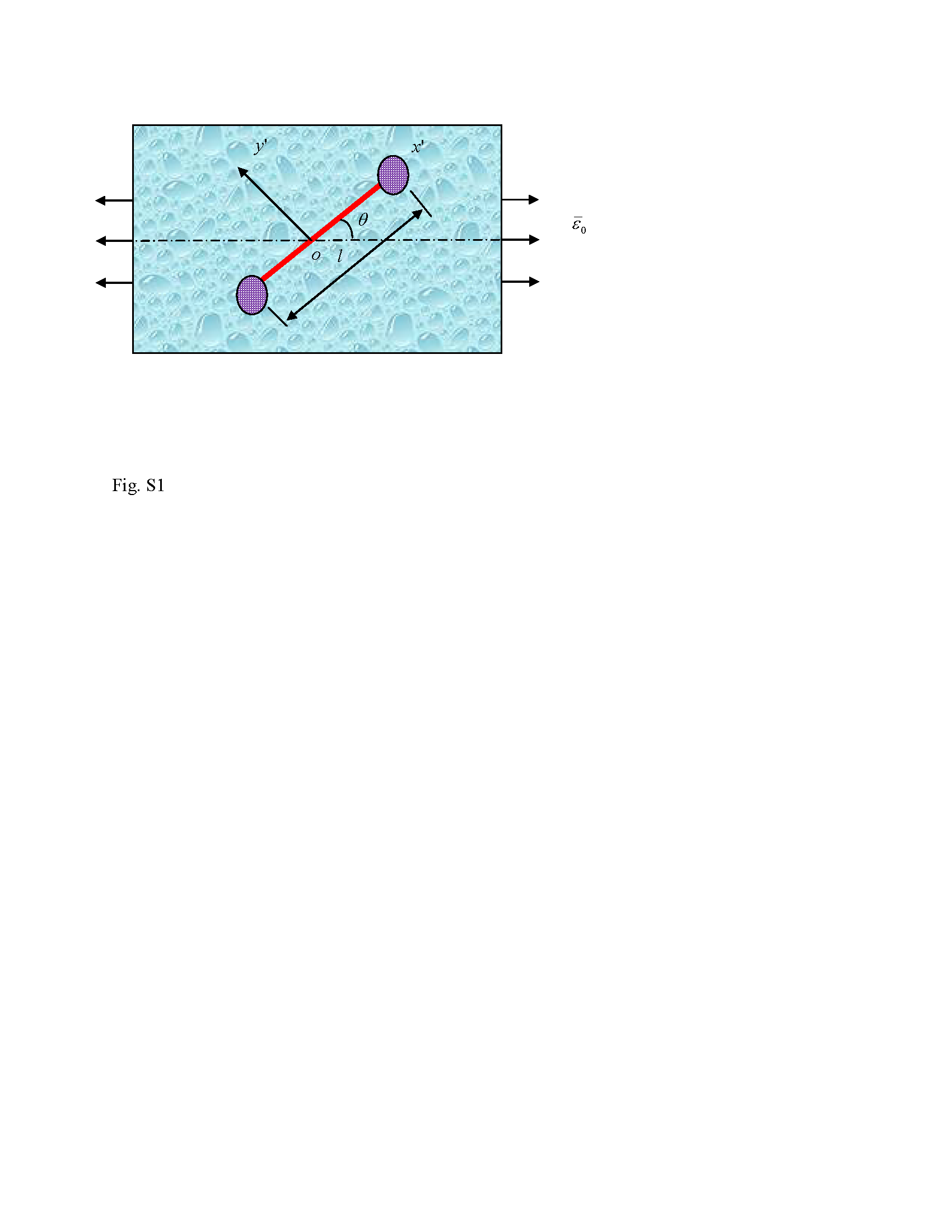

Supplement: Figure S1 — Schematic of a stress fiber at an angle with respect to the direction of cyclic stretch. (TIF) [file pone.0048346.s001.tif]
