# Supplementary material for: Cyclic Stretch Induces Cell Reorientation on Substrates by Destabilizing Catch Bonds in Focal Adhesions
Source: PLoS One. 2012 Nov 12;7(11):e48346. doi: 10.1371/journal.pone.0048346 (PMC3495948; doi:10.1371/journal.pone.0048346)
Supplement: Appendix S1 — Dependence of cyclic stretch amplitude of the SF on the orientation angle. (DOCX) [file pone.0048346.s002.docx]

**Appendix S1 Variation of cyclic stretch amplitude of the SF with the orientation angle**

Figure S1 shows a stress fiber (SF) of length *l* adhering on a substrate. The substrate is subjected to a cyclic uniaxial strain $\bar{\varepsilon}_{0}$. The stress fiber is oriented at an angle $\theta$ with respect to the direction of stretch. In the local coordinates $x'oy'$, where the $x'$ axis coincides with the SF, the tensile strain along the $x'$ direction is

$\varepsilon_{x'x'}=\frac{\left(1-v\right)+(1+v)\cos2\theta}{2}\bar{\varepsilon}_{0}$, (S1)

$v$ being the Poisson’s ratio, and the shear strain is

$\gamma_{x'y'}=-(1+v)\bar{\varepsilon}_{0}\sin2\theta$. (S2)

The cyclic stretch amplitude of the SF is then

, (S3)

where $0<\beta<1$ reflects the effect of elastic anisotropy of the stress fibers on the contribution from the shearing component.
